# Traction method versus conventional endoscopic submucosal dissection for gastric epithelial neoplasms: A randomized controlled trial

**DOI:** 10.1097/MD.0000000000029172

**Published:** 2022-04-01

**Authors:** Jun Kinoshita, Mikitaka Iguchi, Takao Maekita, Ke Wan, Toshio Shimokawa, Kazuhiro Fukatsu, Daisaku Ito, Shinya Taki, Masayuki Nishimoto, Masaki Takao, Yasuto Tabata, Yousuke Mukai, Masayuki Kitano

**Affiliations:** a Second Department of Internal Medicine, Wakayama Medical University, Wakayama, Japan; b Clinical Support Center, Wakayama Medical University, Wakayama, Japan; c Department of Gastroenterology, Wakayama Rousai Hospital, Wakayama, Japan.

**Keywords:** early gastric cancer, EndoTrac, ESD, gastric epithelial neoplasm, traction

## Abstract

**Introduction:**

Endoscopic submucosal dissection (ESD) is an advanced therapeutic technique for en bloc resection of superficial gastrointestinal neoplasms. Although gastric ESD is minimally invasive and provides favorable outcomes, it is technically difficult and requires a long procedure time for dissection. The traction-assisted approach overcomes some of the difficulties of gastric ESD, but its ability to reduce the procedure time remains unclear. The traction-assisted approach using dental floss and a clip did not reduce procedure time in the total population, but it reduced procedure time for lesions limited to the greater curvature of the upper or middle of the stomach. Although the traction direction of the clip-with-line method may be limited to the oral side via the cardia, EndoTrac ESD may provide flexible traction at any time during the procedure. This prospective randomized control study has been designed to compare the efficacy and safety of EndoTrac and conventional gastric ESD.

**Methods/design:**

This multicenter, randomized control trial will enroll 150 patients at 2 hospitals in Japan undergoing EndoTrac or conventional ESD for gastric epithelial neoplasia. Patients with a single gastric epithelial neoplasm who meet the inclusion and exclusion criteria will be randomized to EndoTrac or conventional ESD. Patients will be randomized by a computer-generated random sequence with stratification by operator experience, tumor size, tumor location, and institution. The primary endpoint will be ESD procedure time, defined as the time from the start of the submucosal injection to the completion of resection. Other outcomes will include the rates of adverse events and pathological curability

**Discussion:**

The ability of EndoTrac ESD to reduce the long procedure time and/or adverse events observed with conventional ESD can not only reduce physical stress on the patient, but can also reduce length of hospital stay and medical costs. Reduced technical difficulty will contribute to the widespread adoption of this ESD technique worldwide.

**Trial registration:**

University Hospital Medial Information Network Clinical Trials Registry (UMIN-CTR), ID: 000044450; Registered on June 6, 2021.

https://upload.umin.ac.jp/cgi-open-bin/ctr/ctr_view.cgi?recptno=R000050485.

**Protocol version number:**

1.1, March 1, 2022. Patient enrolment began on June 6, 2021 and is expected to be completed by July 19, 2025.

## 1 Introduction

Endoscopic submucosal dissection (ESD) is an advanced therapeutic technique for en bloc resection of superficial gastrointestinal neoplasms. This technique is minimally invasive and provides patients with favorable outcomes.^[1–6]^ Although developments in devices and techniques have improved outcomes for patients, gastric ESD is difficult to perform because of its complex technique and the long duration. In particular, maintaining an appropriate view of the correct submucosal layer requiring dissection depends on the location of the lesion. For example, tumors located in the greater curvature of the upper or middle third of the stomach easily sink under water due to gravity, whereas the submucosal layer collapses due to the weight of the tumor itself. Thus, tumor location is an important factor associated with prolonged operation time and difficulty in performing gastric ESD.^[7,8]^

Access to the submucosal layer during gastric ESD may be enhanced by various traction devices.^[9–16]^ Although these devices have contributed to overcoming technical difficulties, the ability of traction to reduce procedure time remains unclear. For example, 1 report comparing conventional ESD with traction-assisted ESD using dental floss and hemoclips suggested that traction devices did not reduce procedure time in the total population, but significantly reduced procedure time for gastric neoplasms located in the greater curvature of the upper or middle third of the stomach, without increasing the rate of adverse events (AEs).^[17]^ Thus, the traction direction of the clip-with-line method for gastric ESD may be limited to the oral side via the cardia. Vertical traction to lesions in the greater curvature of the upper or middle third of the stomach may be able to maintain appropriate traction force to dissect the submucosa.

**Figure 1:**
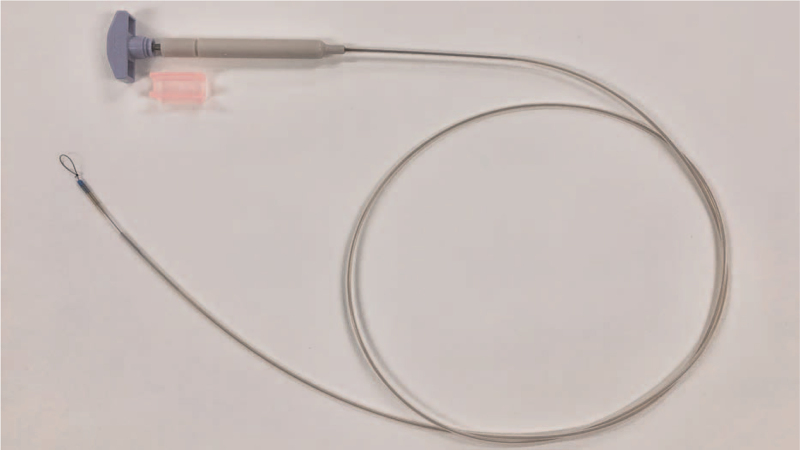
Photograph of the EndoTrac device. This device is composed of a line with a clinch-knotted loop at a tip that passes through a plastic sheath. There is a T-shaped handle at the end.

The EndoTrac traction device (Top Corporation, Tokyo, Japan) is made of a looped nylon thread, a plastic sheath, and a handle (Fig. 1). Pulling the handle pushes the plastic sheath closer to the leading edge of the tumor. The loop is subsequently closed, and the clip is attached to the device. This enables the lesion to be pulled or pushed easily, providing sufficient traction. These findings suggested that the EndoTrac ESD could provide flexible traction at any time during the procedure. To test this hypothesis, a prospective randomized controlled trial (RCT) was designed to test whether EndoTrac traction-assisted ESD provides better procedure-related outcomes than conventional ESD among patients with superficial gastric neoplasms.

## 2 Methods/design

### 2.1 Ethics approval and patient consent

The study protocol was approved by the Wakayama Medical University Ethics Committee (No. 2958), and conformed to the tenets of the Declaration of Helsinki and Ethical Guidelines for Medical and Health Research involving Human Subjects. The trial has been registered with the University Hospital Medical Information Network (trial registration no. UMIN000044450). Written informed consent will be obtained from all patients enrolled in the study.

### 2.2 Study aims and design

This prospective RCT will enroll patients with gastric epithelial neoplasia undergoing ESD at 2 hospitals in Japan. The trial is designed to determine whether EndoTrac ESD provides better efficacy and safety than conventional ESD in patients with gastric epithelial neoplasia. The primary endpoint will be ESD procedure time.

### 2.3 Patients

**Figure 2:**
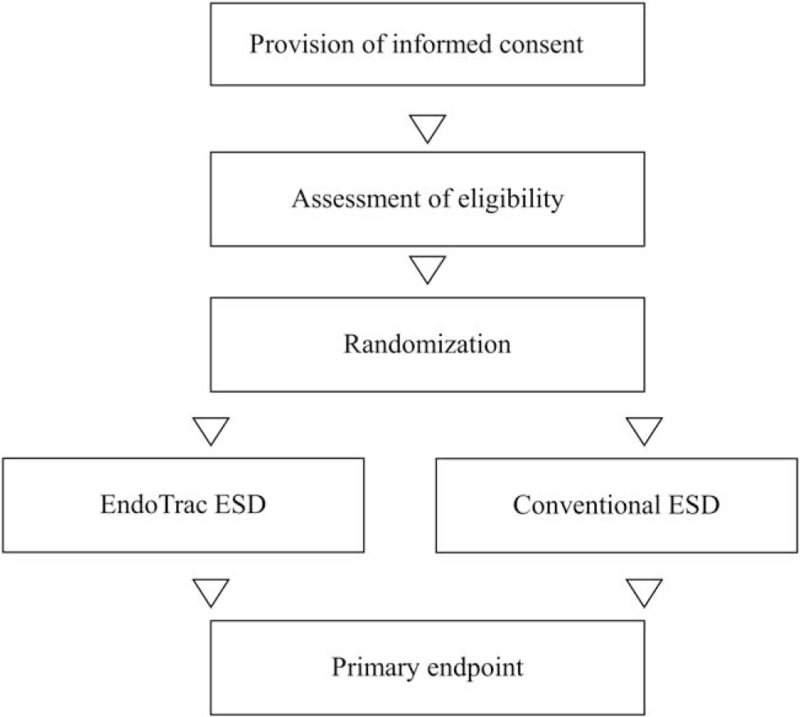
Flow chart of the study design. ESD = endoscopic submucosal dissection.

At each center, the on-site study investigators will obtain informed consent from candidates. An electronic data capture system (University Hospital Medial Information Network Internet Data and Information Center for Medical Research Cloud) will be used to input necessary information, confirm that the candidates meet the eligibility criteria (i.e., that the candidates meet all the inclusion criteria and none of the exclusion criteria). The candidates will be registered, and each included candidate will be assigned a registration number. Patients who complete registration will be randomized 1:1 using the minimization method to undergo EndoTrac or conventional ESD using 4 stratification factors: operator experience, tumor size, tumor location, and institution. A flowchart of the study design is shown in Figure 2.

### 2.4 Inclusion criteria

Patients will be included if they are aged ≥20 years and have a gastric epithelial tumor eligible for ESD in accordance with the 2020 ESD/endoscopic mucosal resection guidelines for gastric cancer, that is, a clinical diagnosis of intra-mucosal cancer (clinical tumor 1a [cT1a]), indicating a differentiated adenocarcinoma of any size, without ulcerative findings; a class cT1a differentiated adenocarcinoma, ≤30 mm in size, with ulcerative findings, or a class cT1a undifferentiated adenocarcinoma, ≤20 mm in size, without ulcerative findings. These criteria are absolute indications for ESD, whereas other criteria are relative indications.^[18]^ For inclusion, patients will also have to have an Eastern Cooperative Oncology Group Performance status ≤2, and be fully informed about and understand the requirements for study participation, and provide voluntary written informed consent.

### 2.5 Exclusion criteria

Patients will be excluded if pre-operative examination shows indications of deep submucosal invasion; the tumor is diagnosed pathologically as not being a gastric epithelial tumor; the tumor cannot be visualized in its entirety; or the tumor crosses the esophagogastric junction or pylorus ring. Patients will also be excluded if they are scheduled to undergo ESD for ≥2 tumors at the same time; have a history of gastrectomy or reconstructive surgery of the gastric tract; have a tendency to bleed (prothrombin time-international normalized ratio >1.5 or platelet count <50,000/μL); are taking 2 or more antiplatelet agents or 1 anticoagulant, with patients taking 1 antiplatelet agent expected to follow appropriate guidelines^[19]^; have serious complications in other organs, defined as an American Society of Anesthesiologists physical status ≥4; are pregnant; or are determined ineligible for any other reason by the principal investigator or sub-investigator.

### 2.6 Randomization and blinding

Patients will be randomly assigned 1:1 to undergo conventional ESD or EndoTrac ESD. Random assignment will be centrally controlled by the Internet Data and Information Center for Medical Research cloud version (INDICE Cloud), supported by University Hospital Medial Information Network. Randomization will be performed via dynamic balancing using the minimization method, with stratification by operator experience (trainee or expert), tumor size (≤20 mm or >20 mm), tumor location (upper/middle thirds or lower third of the stomach), and institution. Neither patients nor physicians will be blinded to patient allocation.

### 2.7 ESD procedures

Following detection of the lesion, several marking dots will be made outside the lesion, solutions will be injected into the submucosa and circumferential incision and submucosal dissection will be continued to completion. The use of any endoscope, injection needle, hemostatic forceps, or hoods will be permitted, but only a Dual Knife (Olympus) will be allowed for circumferential incision and submucosal dissection. The number of EndoTracs used per patient in the EndoTrac ESD group will be recorded. If a snare technique is required, it can be used without being regarded as a protocol deviation, and this patient will be recorded as undergoing hybrid ESD. Biopsies of other lesions will be allowed before or after ESD on the same day. To ensure the safety of patients during ESD procedures performed by trainees, an expert will be available to continue the procedure when the expert regards the trainee as unable to complete the procedure. If the procedure is time ≥60 minutes, and continuing the assigned procedure is deemed impossible, conversion to the other procedure will be allowed. That is, use of an EndoTrac will be permitted in patients undergoing conventional ESD, and removal of the EndoTrac will be permitted in patients undergoing EndoTrac ESD.

#### 2.7.1 Criterion of operator

In this study, the operator must be a research investigator or coordinator who has performed more than 5 ESD procedures for gastric lesions, and at least 1 ESD procedure with an EndoTrac.

#### 2.7.2 Conventional ESD procedure

After marking around the lesion with an electrosurgical knife and submucosal injection, submucosal dissection will be performed without any traction in principle, and the lesion will be removed.

#### 2.7.3 EndoTrac ESD procedure

After marking around the lesion with an electrosurgical knife and submucosal injection, circumferential incisions will be made and the submucosa will be trimmed at the edge of the incision. The endoscope will be withdrawn to set up the EndoTrac procedure. A reusable delivery/development catheter with a hemoclip will be inserted through the accessory channel of the endoscope. After the loaded clip is opened, the nylon thread loop of the EndoTrac will be hooked to the clip, the stopper of the hand control unit will be removed, t-shaped handle will be pulled to shrink the loop, and the EndoTrac will be fixed to the clip. The operator will place the endoscope and EndoTrac at the gastric lesion, and the clip will be fired with the EndoTrac to the edge of the lesion for traction. The anchoring sites of the clip will depend on whether the scope position is straight or retroflex. During submucosal dissection, the lesion can be pulled or pushed freely with the hand control unit for appropriate view and traction. Dissection will be continued and the lesion removed.

### 2.8 Primary endpoint

The primary endpoint will be ESD procedure time, defined as the time from the start of submucosal injection to the end of tumor removal. If an expert replaces a trainee during the procedure, the time required by the expert will be included in the procedure time for the trainee.

### 2.9 Secondary endpoints

Secondary endpoints will include ESD procedure time according to the proficiency of the operator (trainee or expert), tumor size (≤20 mm or >20 mm), tumor location, the presence of an ulcer (UL) (positive or negative), and institution (high volume or low volume center).

Operators who have performed ≤40 ESD procedures previously will be considered trainees, and those who have performed >40 ESD procedures will be considered experts.^[2,7,8,20–22]^ Tumor location will be defined as the affected portion of the stomach (upper, middle, or lower third) or the surface of the stomach (lesser curvature, greater curvature, anterior wall, or posterior wall), according to the Japanese classification of gastric carcinoma.^[23]^ Findings consistent with an UL will be evaluated pathologically, with the presence of an UL or UL scar defined as UL-positive. Institutions performing ≤120 and >120 gastric ESDs per year will be classified as low and high volume centers, respectively.

Also evaluated will be the time for equipping the EndoTrac, defined as the time from insertion of the reusable delivery/development catheter with a hemoclip through the accessory channel of the endoscope to the time when the clip with the EndoTrac is attached to the lesion for traction, and the time from starting traction with the EndoTrac to complete resection. Other secondary outcomes will include the clip slip-off rate during EndoTrac ESD, defined as the number of times a clip slips off the lesion before the end of the procedure; the number of EndoTracs used per patient; and the conversion rate between conventional and EndoTrac ESD.

The rate of damage to the specimen will also be evaluated, and defined in the absence of the EndoTrac as damage within the specimen area delimited by the marked points, or traction-related damage caused by movement of the EndoTrac or the related endoscope within the specimen area delimited by the marked points.

Histologic outcomes will include en bloc, R0, and curative resection rates, tumor depth, sizes of the specimens and tumors, histologic type, ulcerative findings, and margin involvement. En bloc resection will be defined as removal of the entire tumor as a single piece; R0 resection as en bloc resection with lateral and vertical margins free of tumor cells; and curative resection as pathologically eCura A or eCura B, as defined by the Japanese gastric cancer treatment guidelines 2018.^[18]^

AEs will include post-ESD bleeding, clinical symptoms, need for emergency endoscopy, perforation, and pneumonia. Post-ESD bleeding will be defined as hemorrhage with clinical symptoms, confirmed by emergency endoscopy, from the time of the completion of ESD until the end of the protocol. Clinical symptoms will include hematemesis, melena or a >2 g/dL reduction in hemoglobin concentration since the patient's most recent laboratory test. An emergency endoscopy will be defined as an endoscopy performed on a patient who experienced clinical symptoms with suspicion of post-ESD bleeding. Patients who underwent hemostasis for subclinical bleeding without any suspicion of post-ESD bleeding during second look endoscopy will not be regarded as experiencing post-ESD bleeding.^[24]^ Perforation will be defined as mesenteric fat or the intra-abdominal space observed during the procedure or free air found on radiography or computed tomography. Pneumonia after ESD will be defined as clinical findings suggesting pneumonia and radiographic or computed tomography evidence of pneumonia.

### 2.10 Data collection

**Figure 3:**
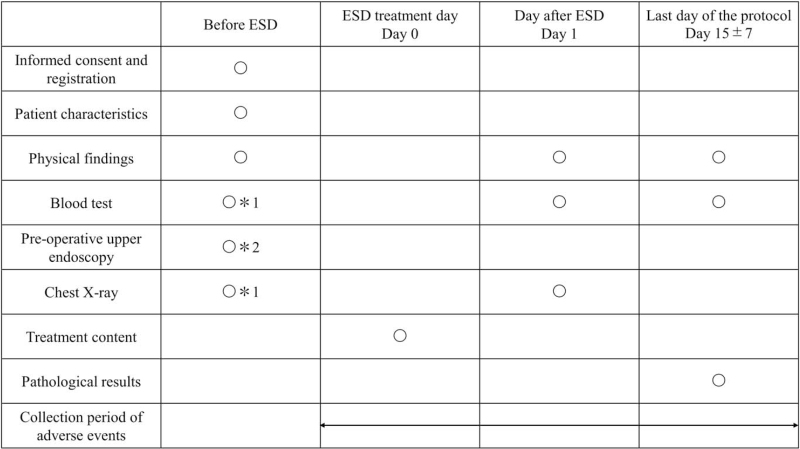
Schedule for data collection. ∗1: within 28 days before registration. ∗2: within 120 days before registration. ESD = endoscopic submucosal dissection.

Baseline assessment before ESD will include patient sex, date of birth, date of obtaining informed consent, and history of gastrectomy or reconstructive surgery of the gastric tract, assessment of performance status, American Society of Anesthesiologists physical status, and use of antithrombotic agents. Other assessments will include subjective findings; objective findings; and the results of hematological examinations, blood coagulation tests, upper endoscopy, and X-ray examination of the chest. Treatment parameters will be assessed on the day of ESD, and AEs will be recorded from the day of ESD to discharge 15 ± 7 days later. Physical findings and the results of blood tests and X-ray examination of the chest will be evaluated the day after ESD. Parameters assessed at discharge will include physical findings, blood examination results, and pathological results. The schedule for data collection is shown in Figure 3.

## 3 Statistical analysis

### 3.1 Sample size calculation

Because this will be a superiority study, the sample size will be estimated based on the primary endpoint (i.e., ESD procedure time). A single center retrospective study compared a traction ESD method using dental floss and a hemoclip in 53 patients with conventional ESD in 185 patients, including propensity score matching analysis.^[25]^ That study found that the ESD procedure time was significantly shorter for the traction than for the conventional ESD group (82.2 ± 79.5 minutes vs 118.2 ± 71.6 minutes, respectively; *P* = .002).^[25]^

Calculations based on these findings showed that 142 patients were needed to ensure 80% power for a 2-sided significance level of 0.05. Assuming a dropout rate of 5%, a sample size of 150 patients has been planned.

### 3.2 Statistical analysis

The primary endpoint is procedure time, defined as the time from the start of local injection into the submucosal layer to the end of detachment. The multiple regression analysis will be performed to estimate the treatment effect between groups adjusted with the stratified factors (i.e., operator proficiency, tumor size, institution and lesion location).

The secondary endpoint includes the factors associated with ESD procedure time which consist of operator proficiency (trainee vs expert), tumor size (≤20 mm vs ≥20 mm), lesion location, findings associated with UL (positive vs negative), and institution (high volume center vs low volume center); EndoTrac equipping time; time from starting traction with EndoTrac to complete resection; clip slip-off rate during EndoTrac and number of EndoTrac used; conversion rate; rate of specimen damage; histologic findings; and AE. The continuous outcomes will be summarized into the mean and standard error. When these outcomes have non-normal distribution, will be used the median and interquartile range as summary statistics. The categorical values will be summarized into frequency (proportion).

## 4 AE reporting

### 4.1 Definition of AE

An AE will be defined as any unfavorable or unintended illness or disability and its manifestations in a subject, regardless of whether it is casually related to the protocol treatment. If an AE is observed in a subject, then the principal investigator (or sub-investigator) will immediately ensure the safety of the subject, take appropriate measures, and describe the event in the case report. In this study, AEs will be reported from the date of obtaining informed consent to the end of the protocol 15 ± 7 days after ESD.

Post-ESD bleeding, perforation, and pneumonia after ESD will be assessed as defined above. AEs and reactions will be evaluated using American Society of Anesthesiologists guidelines^[26]^ and Common Terminology Criteria for Adverse Events v5.0 criteria.

### 4.2 Serious AEs

Serious AEs will be defined as AEs occurring during or at any time after the procedure that results in death; is immediately life-threatening; requires in-patient hospitalization or prolongation of current hospitalization; results in persistent or significant disability or incapacity; or results in a congenital abnormality or birth defect.

The principal investigator at the institution where the serious AE occurs will take appropriate measures, regardless of whether or not there is a causal relationship between the AE and the treatment protocol. The investigator will immediately report the details of the event to the head of the research institution and the principal investigator of the study, in accordance with the regulations of the respective medical institution.

### 4.3 Monitoring

Central monitoring will be performed once per year by an independent data monitoring committee. The monitoring committee will collect information on the status of accumulation, inclusion/exclusion criteria, serious AEs, and any other relevant information, and strive to provide feedback to participating institutions for early resolution if there are any problems.

## 5 Discussion

Generally, ESD requires a professional endoscopist because of the need to maintain an appropriate view to dissect the submucosal layer, pre-coagulate thick blood vessels, and stop intra-operative bleeding rapidly. Because maintaining an appropriate submucosal view is the key to safe and rapid ESD, maintaining the appropriate tension on the submucosa for dissection using an endoscope hood or gravity is needed. Various traction devices have been developed to visualize the submucosal layer during ESD, thereby overcoming these technical difficulties.^[9–16]^

Ensuring the appropriate direction of traction, however, is important. For example, a recent multicenter RCT found that although procedure times did not differ significantly between patients who underwent the clip-with-line method and those who underwent conventional gastric ESD, the procedure times were shorter using the clip-with-line method for lesions located at the greater curvature of the upper and middle thirds of the stomach.^[17]^ These findings suggest that the traction direction may be limited. The clip-with-line method provides 1-way traction for the oral side through the cardia, enabling vertical traction for lesions located at the greater curvature. For other lesions, however, this method provides distal or proximal side traction and cannot view the appropriate submucosal layer for dissection in some patients.

The EndoTrac consists of a line with a clinch-knotted loop, a plastic sheath, and a T-shaped handle. After the line is tied to an endoclip, the distance between the endoclip and the tip of the plastic sheath can be adjusted by pulling or pushing the handle. Attachment of only the endoclip and the tip of the sheath to the lesion without a line would make it difficult to access the submucosal layer because of interference by the sheath. In the EndoTrac, however, the endoclip is tied to a line, providing greater access to the submucosal layer by pushing the sheath and extending the line.^[27]^

EndoTrac has advantages over clip-with-line traction methods. The EndoTrac can not only control the degree of strength but the direction of traction. Use of the EndoTrac enables coordination of an appropriate direction by pulling or pushing the sheath to achieve the desired traction. The clip-with-line method can pull but not push the lesion, with this 1-way traction resulting in inappropriate or excessive traction, preventing the operator from recognizing the appropriate submucosal layer for dissection.

To the best of our knowledge, the study described herein will be the first prospective randomized control study to compare the efficacy and safety of EndoTrac ESD with that of conventional ESD for gastric tumors. Use of a randomized controlled design can reduce biases associated with retrospective studies, such as patient selection, operator selection, small sample size, and the unconscious choice to use EndoTrac.

Factors associated with technical difficulties and long procedure times for gastric ESD include larger lesion sizes, lesions located in the middle and upper two-thirds of the stomach, lesions located in the greater curvature, presence of ulceration, and an operating physician who has performed <40 of these procedures.^[2,7,8,20–22]^ To determine whether these factors are also associated with greater difficulties in patients undergoing EndoTrac ESD, we plan to randomize patients to undergo EndoTrac or conventional ESD by dynamic balancing using the minimization method, and with stratification by operator experience (trainee vs expert), tumor size (≤20 mm vs >20 mm), tumor location (upper/middle thirds vs lower third), and institution (high vs low volume center). The major limitation of this study is that the operator is not blinded to treatment group allocation.

## 6 Conclusions

This study is designed to assess whether EndoTrac ESD is superior to conventional ESD, thereby overcoming the technical difficulties of the latter. This study should resolve clinical questions of whether EndoTrac ESD is more effective and safer than conventional ESD.

## Author contributions

JK, MI, TM, and MK conceived the study, designed the study protocol, and drafted the manuscript. JK and MI wrote the manuscript. KF, DI, ST, MN, MT, YT, and YM are in charge of coordination and direct implementation. KW and TS helped to develop the study measures and analyses. All authors contributed to drafting this study protocol manuscript and have read and approved the final version.

**Conceptualization:** Jun Kinoshita, Mikitaka Iguchi, Takao Maekita, Masayuki Kitano.

**Data curation:** Mikitaka Iguchi, Takao Maekita, Kazuhiro Fukatsu, Daisaku Ito, Shinya Taki, Masayuki Nishimoto, Masaki Takao, Yasuto Tabata, Yousuke Mukai.

**Formal analysis:** Ke Wan, Toshio Shimokawa.

**Investigation:** Mikitaka Iguchi, Takao Maekita, Kazuhiro Fukatsu, Daisaku Ito, Shinya Taki, Masayuki Nishimoto, Masaki Takao, Yasuto Tabata, Yousuke Mukai.

**Methodology:** Mikitaka Iguchi.

**Writing – original draft:** Jun Kinoshita.

**Writing – review & editing:** Mikitaka Iguchi.
